# Butylphthalide reduces plaque burden and improves neurological function in carotid atherosclerotic disease: a pooled analysis

**DOI:** 10.3389/fphar.2025.1460338

**Published:** 2025-03-28

**Authors:** Jia Yang, Xinhao Pei, Jianxin Qiang, Lijuan Li, Yanping Liu, Panpan Hao

**Affiliations:** ^1^ 120 First-aid call center, Wuzhong People's Hospital Affiliated to Ningxia Medical University, Wuzhong, Ningxia, China; ^2^ Department of Cardiology, Qilu Hospital of Shandong University, Jinan, Shandong, China; ^3^ Department of Cardiology, Wuzhong People’s Hospital Affiliated to Ningxia Medical University, Wuzhong, Ningxia, China; ^4^ Department of Radiology, Qilu Hospital of Shandong University, Jinan, Shandong, China

**Keywords:** butylphthalide, carotid, atherosclerosis, intima-media thickness, stroke

## Abstract

**Background:**

The influence of butylphthalide on atherosclerotic plaque burden remains underexplored. This pooled analysis was aimed to evaluate its efficacy and safety in carotid atherosclerosis.

**Methods:**

The literature were retrieved in online databases. Carotid intima-media thickness (IMT), plaque size, Crouse score, National Institute of Health Stroke Scale (NIHSS), circulating biomarkers, and drug-related adverse events were extracted and compared between the butylphthalide group and the control group without butylphthalide.

**Results:**

Nine randomized controlled trials with 892 subjects were included. Compared with the control group, butylphthalide significantly reduced carotid IMT (MD -0.24 mm, 95% CI [-0.31, −0.16], P < 0.00001), plaque size (MD -3.83 mm^2^, 95% CI [-5.64, −2.01], P < 0.0001), Crouse score (MD -0.48, 95% CI [-0.89, −0.08], P = 0.02), hs-CRP (MD -1.65 mg/L, 95% CI [-2.99, −0.30], P = 0.02) and MMP-9 (MD -12.29 μg/L, 95% CI [-16.24, −8.33], P < 0.00001). Neurological improvement (NIHSS reduction: MD -2.94, 95% CI [-4.15, −1.73], P < 0.00001) and comparable safety profiles (OR 0.93, 95% CI [0.37, 2.37], P = 0.89) were observed.

**Conclusion:**

Butylphthalide treatment reduces carotid plaque burden, improves neurological recovery and has a high safety profile, supporting its role in stroke prevention.

## Graphical Abstract

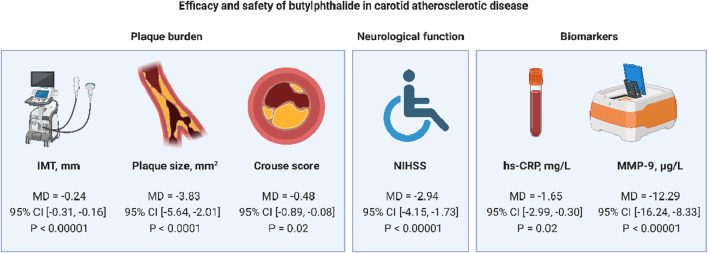

## Highlights

• Butylphthalide significantly reduces carotid plaque burden and improves neurological function.
• Anti-inflammatory and matrix-stabilizing mechanisms underlie its therapeutic effects.
• Butylphthalide should be recommended in the primary and secondary prevention of ischemic stroke.

## 1 Introduction

Carotid atherosclerotic disease is a critical contributor to ischemic stroke. Current therapies, including statins and revascularization, are inadequate for plaque stabilization and neurological recovery. (Sarraju and Nissen, 2024). Dl-3-n-butylphthalide (butylphthalide) is a synthetic racemic 3-n-butylphthalide and the first new drug with independent intellectual property rights for the treatment of cerebrovascular diseases in China. (Chen et al., 2019). Mechanistically, it improves cerebral microcirculation, reduces oxidative stress, restores mitochondrial dysfunction, regulates energy metabolism and inhibits neuronal apoptosis. (Wang et al., 2018; Liu et al., 2007; Que et al., 2021; Zhang et al., 2022). Since 2002, butylphthalide has been approved by the China Food and Drug Administration for clinical trials in the treatment of cerebral ischemia. The 2023 Chinese Stroke Association guidelines for ischemic stroke management recommend butylphthalide as an adjunctive therapy for acute ischemic stroke due to its neuroprotective properties. (Liu et al., 2023). Specifically, the guidelines highlight its role in improving neurological recovery by improving microcirculation and reducing oxidative stress (Class IIa recommendation). (Liu et al., 2023). Recent meta-analyses further support the efficacy of butylphthalide in reducing National Institute of Health Stroke Scale (NIHSS) scores and improving functional outcomes. (Wang et al., 2025; Wang et al., 2022).

While the neurological benefits of butylphthalide are well-documented, its direct impact on the burden of atherosclerotic plaques is unexplored. (Wang et al., 2018). Guidelines remain silent on the evidence for the benefit of butylphthalide on carotid plaque burden, underscoring the novelty of this study. Given the inadequacies of current treatments for carotid stenosis, research into new treatment modalities has become very important. This study addresses two gaps: (1) the lack of pooled evidence on butylphthalide’s impact on carotid plaque burden and (2) its dual role in plaque regression and neurological recovery. By synthesizing RCT data, we provide mechanistic and clinical insights to develop stroke prevention strategies.

## 2 Methods

### 2.1 Database sources and search strategy

We conducted this pooled analysis according to the PRISMA guideline and the Cochrane Handbook version 6.4 (PROSPERO registration number: CRD42024498276). (Page et al., 2021; Higgins et al., 2023). A systematic search was conducted in PubMed, EMBASE, Cochrane Library, Google Scholar and Chinese databases (Chinese Biomedicine Literature Database, China National Knowledge Infrastructure, Chinese Scientific Journal and Wanfang Database) using the keywords: (“butylphthalide” OR “butyl phthalide”) AND (“plaque” OR “atherosclerosis” OR “atheroma”). No language/date restrictions were applied. Additional trials were identified via ClinicalTrials.gov, Chinese Clinical Trial Registry (ChiCTR) and reference screening.

### 2.2 Inclusion and exclusion criteria

Inclusion criteria:

  1) Randomized controlled trials (RCTs) with parallel or crossover design.
2) Comparison of butylphthalide (oral or intravenous) with placebo or standard treatment.
3) Reported outcomes: carotid intima-media thickness (IMT), plaque size, plaque Crouse score, NIHSS, biomarkers such as high-sensitivity C-reactive protein (hs-CRP) and matrix metalloproteinase-9 (MMP-9), and/or adverse events.
4) Minimum follow-up period of 4 weeks.

Exclusion criteria:

  1) Non-randomized studies, case reports, or reviews.
2) Studies using butylphthalide in combination with unapproved experimental therapies.
3) Duplicate publications or incomplete outcome data. If the same study was reported in two or more publications, the one with the most comprehensive information was considered.

### 2.3 Data extraction and quality assessment

Two reviewers independently extracted data using a standardized form, including the year of publication, study design, sample size, patient demographics, intervention details, and outcomes. Risk of bias was assessed using the Cochrane Risk of Bias Tool (RoB 2.0), which evaluates randomization, allocation concealment, blinding, outcome reporting, and dropout rate. Disagreements were resolved by consensus.

### 2.4 Statistical analysis

The RevMan 5.4.1 software was used for the pooled analyses. Heterogeneity was quantified using the I^2^ statistic. Fixed-effects models were applied if I^2^ ≤ 50%, otherwise random-effects models were used. Subgroup analyses stratified by age and treatment duration were performed. Outliers were excluded from sensitivity analyses to assess robustness.

## 3 Results

### 3.1 Characteristics of the included studies

**FIGURE 1:**
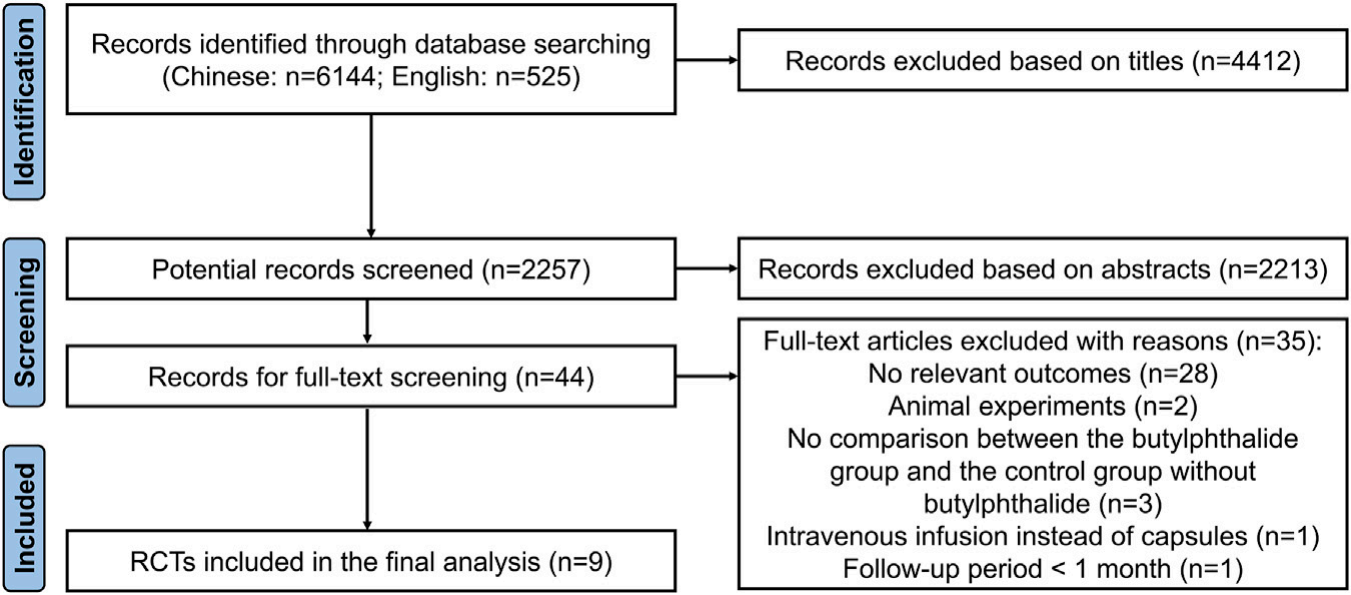
Study diagram of searching and including literature.

Of 6,669 records, nine RCTs (892 patients) met the inclusion criteria (Figure 1). (Li et al., 2021; Sun et al., 2021; Zhang et al., 2020; You et al., 2019; Li et al., 2011; Wang et al., 2017; Wang and Zhang, 2021; Qiu et al., 2018; Lin et al., 2022) The baseline characteristics of the eligible studies are listed in Supplementary Table 1, and the quality assessment is shown in Supplementary Figure 1. The risk of bias assessment indicated moderate quality with concerns about blinding and allocation concealment. There were no incomplete results or selective reporting of results in the nine studies. No study mentioned allocation concealment.

### 3.2 Plaque burden outcomes

**FIGURE 2:**
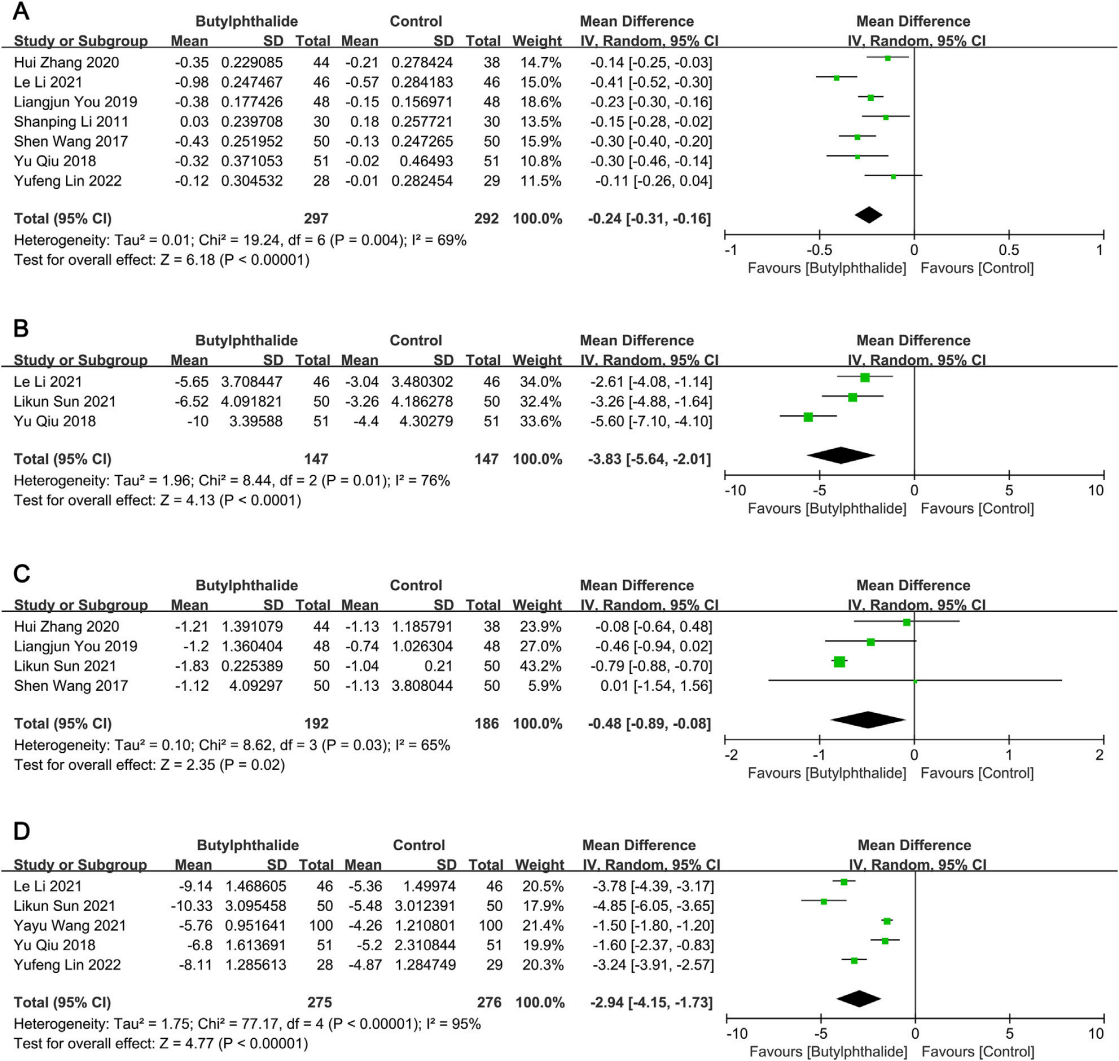
The pooled effects of butylphthalide on carotid plaque burden and neurological function. **(A)** Effect of butylphthalide on carotid intima-media thickness (IMT); **(B)** effect of butylphthalide on carotid plaque size; **(C)** effect of butylphthalide on plaque Crouse score; **(D)** effect of butylphthalide on the National Institute of Health Stroke Scale (NIHSS).

Compared with the control group, the reductions from baseline to the end of the follow-up period in carotid IMT (mean difference [MD] −0.24 mm, 95% confidence interval [CI] [-0.31, −0.16], P < 0.00001; Figure 2A), plaque size (MD -3.83 mm^2^, 95% CI [-5.64, −2.01], P < 0.0001; Figure 2B) and plaque Crouse score (MD -0.48, 95% CI [-0.89, −0.08], P = 0.02; Figure 2C) were significantly greater in the butylphthalide group.

There was significant heterogeneity for the above outcomes among the included studies, therefore subgroup analyses were performed. Heterogeneity in IMT was partially explained by variability in mean age (≥60 years: I^2^ = 86% and P = 0.0009 for heterogeneity; <60 years: I^2^ = 33% and P = 0.23 for heterogeneity) and treatment duration (≥6 months: I^2^ = 48% and P = 0.11 for heterogeneity; <6 months: I^2^ = 17% and P = 0.27 for heterogeneity), and the results were in favor of the butylphthalide groups in all subgroups (all P < 0.05) (Table 1). For plaque size, deletion of the study with patients with mean age ≥60 years did not reverse the results (MD -4.45 mm^2^, 95% CI [-6.74, −2.16], P = 0.0001), (Li et al., 2021), but for plaque Crouse score, exclusion of the study with treatment duration <6 months reversed the result (MD -0.28, 95% CI [-0.64, 0.07], P = 0.12) (Table 1). (Sun et al., 2021) Thus, butylphthalide treatment significantly reduced carotid IMT, plaque size and plaque Crouse score. Subgroup analyses revealed that the reductions in IMT and plaque size were consistent across subgroups, but the improvement in Crouse score depended on the duration of treatment.

**TABLE 1:** Subgroup analyses of the efficacy and safety of butylphthalide in patients with carotid atherosclerotic disease.

Outcome	References	Patients, n	Mean difference/Odds ratio, 95% CI	P value	I^2^, %	Heterogeneity P value
**IMT, mm**	12,14–17,19,20	589	−0.24 [-0.31, −0.16]	<0.00001	69	0.004
**Adjustment for mean age, years**						
≥60	12,16,20	209	−0.23 [-0.42, −0.03]	0.02	86	0.0009
<60	14,15,19	280	−0.22 [-0.27, −0.16]	<0.00001	33	0.23
**Adjustment for treatment and follow-up period, months**						
≥6	14–17,20	395	−0.21 [-0.25, −0.17]	<0.00001	48	0.11
<6	12,19	194	−0.38 [-0.47, −0.29]	<0.00001	17	0.27
**Plaque size, mm** ^**2**^	12,13,19	294	−3.83 [-5.64, −2.01]	<0.0001	76	0.01
**Adjustment for mean age, years**						
≥60	12	92	−2.61 [-4.08, −1.14]	0.0005	NA	NA
<60	13,19	202	−4.45 [-6.74, −2.16]	0.0001	77	0.04
**Plaque Crouse score**	13–15,17	378	−0.48 [-0.89, −0.08]	0.02	65	0.03
**Adjustment for treatment and follow-up period, months**						
≥6	14,15,17	278	−0.28 [-0.64, 0.07]	0.12	0	0.56
<6	13	100	−0.79 [-0.88, −0.70]	<0.00001	NA	NA
**NIHSS**	12,13,18–20	551	−2.94 [-4.15, −1.73]	<0.00001	95	<0.00001
**Adjustment for mean age, years**						
≥60	12,20	149	−3.54 [-3.98, −3.09]	<0.00001	27	0.24
<60	13,18,19	402	−2.54 [-4.08, −1.00]	0.001	93	<0.00001
**Adjustment for treatment and follow-up period, months**						
≥6	18,20	257	−2.34 [-4.05, −0.64]	0.007	95	<0.00001
<6	12,13,19	294	−3.37 [-5.13, −1.62]	0.0002	93	<0.00001
**hs-CRP, mg/L**	12,14–18	630	−1.65 [-2.99, −0.30]	0.02	95	<0.00001
**Adjustment for mean age, years**						
≥60	12,16	152	−2.66 [-6.63, 1.31]	0.19	97	<0.00001
<60	14,15,18	378	−1.25 [-2.14, −0.36]	0.006	76	0.02
**Adjustment for treatment and follow-up period, months**						
≥6	14–18	538	−0.97 [-1.53, −0.40]	0.0008	60	0.04
<6	12	92	−4.65 [-5.40, −3.90]	<0.00001	NA	NA
**MMP-9, μg/L**	14,15,17	278	−12.29 [-16.24, −8.33]	<0.00001	0	0.96
**Adverse reaction**	12–14,16–18,20	699	0.93 [0.37, 2.37]	0.89	45	0.10
**Adjustment for mean age, years**						
≥60	12,16,20	217	3.86 [0.62, 24.09]	0.15	0	0.78
<60	13,14,18	382	0.45 [0.24, 0.86]	0.02	50	0.13
**Adjustment for treatment and follow-up period, months**						
≥6	14,16–18,20	499	0.49 [0.27, 0.90]	0.02	35	0.20
<6	12,13	200	3.13 [0.62, 15.89]	0.17	0	1.00

IMT, intima-media thickness; NIHSS, national institute of health stroke scale; NA, not applicable.

### 3.3 Neurological and biomarker outcomes

Compared to the control group, the reductions from baseline to the end of the follow-up period in NIHSS (MD -2.94, 95% CI [-4.15, −1.73], P < 0.00001; Figure 2D), hs-CRP (MD -1.65 mg/L, 95% CI [-2.99, −0.30], P = 0.02; Supplementary Figure 2A) and MMP-9 (MD -12.29 μg/L, 95% CI [-16.24, −8.33], P < 0.00001; Supplementary Figure 2B) were significantly greater in the butylphthalide group.

Subgroup analyses revealed that the improvement in NIHSS and MMP-9 with butylphthalide was consistent across subgroups. For hs-CRP, the result favored the butylphthalide group in the younger cohorts (<60 years) (MD -1.25 mg/L, 95% CI [-2.14, −0.36], P = 0.006), but the reduction in hs-CRP was not significant in the older cohorts (≥60 years) (MD -2.66 mg/L, 95% CI [-6.63, 1.31], P = 0.19) (Table 1).

### 3.4 Safety

Drug-related adverse reactions included gastrointestinal complaints, liver damage, headache, dizziness and skin rash. However, no differences were observed between the two groups, indicating comparable safety (odds ratio [OR] 0.93 95% CI [0.37, 2.37], P = 0.89; Supplementary Figure 3). And the heterogeneity among the studies was at the borderline of significance (I^2^ = 45%, P = 0.10). The risk of drug-related adverse reactions was lower in the butylphthalide group than in the control group in the subgroup with a mean age of <60 years and in the subgroup with a treatment duration of ≥6 months, and the risk was similar between the two groups in the subgroup with a mean age of ≥60 years and in the subgroup with a treatment duration of <6 months (Table 1).

## 4 Discussion

We found that the butylphthalide group was more effective than the control group in treating the atherosclerotic plaques in the carotid artery. The NIHSS as a neurological function test was also improved by butylphthalide treatment in patients with ischemic stroke, which was consistent with a previous pooled analysis. (Fan et al., 2022). To date, there has been no pooled analysis of the effect of butylphthalide treatment on carotid plaques. Our study fills this gap and provides more comprehensive and convincing evidence for the benefits of butylphthalide in carotid atherosclerotic disease. More importantly, this study bridges this gap by evaluating butylphthalide’s dual role in plaque regression and neurological improvement, aligning with emerging priorities in stroke prevention.

Butylphthalide has been researched and used clinically for more than 30 years, with a focus on the treatment of ischemic encephalopathy. Currently, it is mainly used for the treatment of ischemic stroke. In recent years, a number of researchers in China have also investigated the effects of butylphthalide on improving cognitive function. (Wang et al., 2025; Wang et al., 2022; Fan et al., 2022). Butylphthalide has been reported to protect neurons and repair neurological injury by inhibiting inflammasome activation, increasing antioxidant activity and preventing mitochondrial damage. (Que et al., 2021). Our findings align with prior studies demonstrating the neuroprotective effect of butylphthalide (e.g., NIHSS reduction: MD -2.94 vs Fan et al., 2022: MD -2.1). (Wang et al., 2025; Wang et al., 2022; Fan et al., 2022). Notably, the NIHSS improvement corroborates prior meta-analyses, extending evidence to carotid atherosclerosis populations. There are also clinical trials investigating the effect of butylphthalide on the progression and stability of carotid atheroma plaques. However, these are small-sample clinical trials and no pooled analysis has been performed to test this question. (Li et al., 2021; Sun et al., 2021; Zhang et al., 2020; You et al., 2019; Li et al., 2011; Wang et al., 2017; Wang and Zhang, 2021; Qiu et al., 2018; Lin et al., 2022). Our study demonstrates butylphthalide’s dual efficacy in reducing carotid plaque burden and improving neurological function. Plaque regression (IMT: MD -0.24 mm) parallels reductions in hs-CRP and MMP-9, suggesting anti-inflammatory and matrix-stabilizing effects. The following hypotheses are proposed as possible mechanisms for the therapeutic effect of butylphthalide on the progression and stability of carotid plaques: 1) Butylphthalide has the potential to decrease the expression and deposition of MMPs and the amyloid precursor protein β-amyloid 40 (Aβ40); (Wei et al., 2012); 2) Butylphthalide inhibits PDGF-BB-stimulated proliferation of vascular smooth muscle cells by inducing autophagy; (Hu et al., 2016); 3) Butylphthalide inhibits inflammatory responses and oxidative stress by regulating NF-κB, p38-MAPK, HIF-1α and AMPK/SIRT1 signaling pathways. (Zhang et al., 2022).

Compared to statins, the cornerstone of plaque stabilization, butylphthalide uniquely combines plaque regression with neuroprotection, supporting its complementary role in stroke prevention. However, heterogeneity in older subgroups (I^2^ = 86%) suggests age-dependent efficacy, possibly due to comorbid conditions affecting drug response. This study demonstrates that butylphthalide reduces carotid plaque burden and improves neurologic recovery, consistent with its established role in ischemic stroke. The mechanistic plausibility lies in its anti-inflammatory (NF-κB suppression) and antioxidant (AMPK/SIRT1 activation) properties, which stabilize plaques by inhibiting MMP-9 and smooth muscle proliferation. (Zhang et al., 2022).

### 4.1 Clinical implications

Butylphthalide offers dual benefits—plaque regression and neuroprotection—making it a candidate for primary/secondary stroke prevention. Current guidelines should consider these findings to expand therapeutic recommendations.

### 4.2 Limitations

This pooled analysis has several limitations. First, the quality of the included articles was not high, and most of them did not use blind methods, which could lead to misclassification. Second, the treatment and follow-up periods were relatively short (<6 months in 44% of studies). Short follow-up periods and variability in blinding limit generalizability. Future RCTs should prioritize standardized protocols, integrate advanced imaging (e.g., plaque neovascularization), and extend observation periods.

## 5 Conclusion

Butylphthalide significantly reduces carotid atherosclerosis progression and improves neurological outcomes, with a favorable safety profile. These findings advocate its inclusion in comprehensive stroke prevention guidelines.

## Data Availability

The original contributions presented in the study are included in the article/Supplementary Material, further inquiries can be directed to the corresponding author.

## Abbreviations

IMT, intima-media thickness; MD, mean difference; NIHSS, National Institute of Health Stroke Scale.

## References

[B1] Chen X. Q. Qiu K. Liu H. He Q. Bai J. H. Lu W. (2019). Application and prospects of butylphthalide for the treatment of neurologic diseases. Chin. Med. J. Engl. 132 (12), 1467–1477. 10.1097/CM9.0000000000000289 31205106 PMC6629339

[B2] Fan X. Shen W. Wang L. Zhang Y. (2022). Efficacy and safety of DL-3-n-butylphthalide in the treatment of poststroke cognitive impairment: a systematic review and meta-analysis. Front. Pharmacol. 12, 810297. 10.3389/fphar.2021.810297 35145408 PMC8823901

[B3] Higgins J. P. T. Thomas J. Chandler J. Cumpston M. Li T. Page M. J. (2023). Cochrane Handbook for Systematic Reviews of Interventions. 2nd Edition. Chichester, United Kingdom: John Wiley and Sons. 10.1002/14651858.ED000142 PMC10284251 31643080

[B4] Hu H. Liu B. Zuo Y. Liu D. Xie R. Cui W. (2016). dl-3-n-butylphthalide suppresses PDGF-BB-stimulated vascular smooth muscle cells proliferation via induction of autophagy. Life Sci. 151, 182–188. 10.1016/j.lfs.2016.03.010 26968782

[B5] Li L. Huang L. Liu J. Chang B. (2021). Effect of butylphthalide combined with Xuesaitong on carotid atherosclerosis and inflammatory factors in patients with acute cerebral infarction complicated with type 2 diabetes mellitus. Hainan Med. J. [in Chinese] 32 (8), 966–969. 10.3969/j.issn.1003-6350.2021.08.004

[B6] Li S. Li H. Wu X. (2011). Effect of butylphthalide on carotid intima-media thickness and high-sensitivity C-reactive protein in elderly hypertensive patients. Chinese Journal of Clinical Pharmacology and Therapeutics [in Chinese] 16 (3), 331–333. Available online at: http://xdyx.bjzzcb.com/xdyx/article/abstract/202115028

[B7] Lin Y. Wang Z. Wang X. (2022). Butylphthalide improves the prognosis of patients with acute cerebral infarction and large-artery atherosclerosis by decreasing serum lipoprotein-related phospholipase A2. Tianjin Medical Journal [in Chinese] 50 (5), 544–547. 10.11958/20212398

[B8] Liu C. L. Liao S. J. Zeng J. S. Lin J. W. Li C. X. Xie L. C. (2007). dl-3n-butylphthalide prevents stroke via improvement of cerebral microvessels in RHRSP. J Neurol Sci 260 (1-2), 106–113. 10.1016/j.jns.2007.04.025 17553527

[B9] Liu L. Li Z. Zhou H. Duan W. Huo X. Xu W. (2023). Chinese Stroke Association guidelines for clinical management of ischaemic cerebrovascular diseases: executive summary and 2023 update. Stroke Vasc Neurol 8 (6), e3. 10.1136/svn-2023-002998 38158224 PMC10800268

[B10] Page M. J. McKenzie J. E. Bossuyt P. M. Boutron I. Hoffmann T. C. Mulrow C. D. (2021). The PRISMA 2020 statement: an updated guideline for reporting systematic reviews. BMJ 372, n71. 10.1136/bmj.n71 33782057 PMC8005924

[B11] Qiu Y. Peng X. Zhang Y. Zeng S. Wen Q. (2018). The effect of butylphthalide on carotid plaque size and neural function in patients with acute cerebral infarction. Chinese Journal of Modern Drug Application [in Chinese] 12 (14), 1–3. 10.14164/j.cnki.cn11-5581/r.2018.14.001

[B12] Que R. Zheng J. Chang Z. Zhang W. Li H. Xie Z. (2021). Dl-3-n-Butylphthalide rescues dopaminergic neurons in Parkinson's disease models by inhibiting the NLRP3 inflammasome and ameliorating mitochondrial impairment. Front Immunol 12, 794770. 10.3389/fimmu.2021.794770 34925379 PMC8671881

[B13] Sarraju A. Nissen S. E. (2024). Atherosclerotic plaque stabilization and regression: a review of clinical evidence. Nat Rev Cardiol 21 (7), 487–497. 10.1038/s41569-023-00979-8 38177454

[B14] Sun L. Zhao F. Chang W. (2021). Efficacy of butylphthalide combined with transcranial ultrasound thrombolysis in patients with carotid atherosclerotic plaques. Guangxi Medical Journal [in Chinese] 43 (10), 1194–1197. 10.11675/j.issn.0253-4304.2021.10.06

[B15] Wang H. Ye K. Li D. Liu Y. Wang D. (2022). DL-3-n-butylphthalide for acute ischemic stroke: an updated systematic review and meta-analysis of randomized controlled trials. Front Pharmacol 13, 963118. 10.3389/fphar.2022.963118 36120291 PMC9479342

[B16] Wang S. Fang Q. Wu X. Zhao Q. Feng X. Yang T. (2017). Effect of butylphthalide on plaque stability and inflammatory in patients with carotid atherosclerosis. Journal of Hainan Medical University [in Chinese] 23 (7), 946–949. 10.13210/j.cnki.jhmu.20170113.007

[B17] Wang S. Ma F. Huang L. Zhang Y. Peng Y. Xing C. (2018). Dl-3-n-Butylphthalide (NBP): a promising therapeutic agent for ischemic stroke. CNS Neurol Disord Drug Targets 17 (5), 338–347. 10.2174/1871527317666180612125843 29895257

[B18] Wang Y. Li M. Jiang Y. Ji Q. (2025). Comparative efficacy of neuroprotective agents for improving neurological function and prognosis in acute ischemic stroke: a network meta-analysis. Front Neurosci 18, 1530987. 10.3389/fnins.2024.1530987 39834702 PMC11743486

[B19] Wang Y. Zhang M. (2021). Efficacy of butylphthalide in patients with cerebral infarction and carotid atherosclerosis. Modern Medicine and Health Research [in Chinese] 5 (15), 70–72. Available online at: http://xdyx.bjzzcb.com/xdyx/article/abstract/202115028

[B20] Wei W. Zhang W. Huang Y. Li Y. Zhu G. Chen F. (2012). The therapeutic effect of (DL)-3-n-butylphthalide in rats with chronic cerebral hypoperfusion through downregulation of amyloid precursor protein and matrix metalloproteinase-2. J Int Med Res 40 (3), 967–975. 10.1177/147323001204000315 22906269

[B21] You L. Zuo X. Cui B. (2019). The effect of butylphthalide on the stability of vulnerable carotid plaque in patients with ischemic cerebrovascular disease. Chinese Journal of Rational Drug Use [in Chinese] 16 (6), 35–37. 10.3969/j.issn.2096-3327.2019.6.011

[B22] Zhang H. Zhang M. Wang L. (2020). Efficacy of butylphthalide in patients with carotid atherosclerosis. Medical Journal of Chinese People's Health [in Chinese] 32 (9), 26–28. 10.3969/j.issn.1672-0369.2020.09.011

[B23] Zhang Y. Ren Y. Chen X. Deng S. Lu W. (2022). Role of butylphthalide in immunity and inflammation: butylphthalide may Be a potential therapy for anti-inflammation and immunoregulation. Oxid Med Cell Longev 2022, 7232457. 10.1155/2022/7232457 35422893 PMC9005281

