# Supplementary material for: Butylphthalide reduces plaque burden and improves neurological function in carotid atherosclerotic disease: a pooled analysis
Source: Front Pharmacol. 2025 Mar 28;16:1460338. doi: 10.3389/fphar.2025.1460338 (PMC11986422; doi:10.3389/fphar.2025.1460338)

**Supplementary Tables**

**Supplementary Table 1. The characteristics of the included studies**

| **Study** | **Subjects** | **Sample size** | **Age, years** | **Mean follow-up duration, months** | **Conventional treatment** | **Outcomes** |
| --- | --- | --- | --- | --- | --- | --- |
| Zhang H, et al. 2020 [14] | Carotid atherosclerosis with acute cerebral infarction | 82 | 59.53±2.61 | 6 | Aspirin and atorvastatin | Carotid IMT; plaque Crouse score; hs-CRP; TNF-α; MMP-9; adverse reactions |
| Li L, et al. 2021 [12] | Carotid atherosclerosis with acute cerebral infarction and type 2 diabetes | 92 | 64.99±4.35 | 1 | Antiplatelet aggregation and lipid-lowering agents; Xuesaitong tablets | Carotid IMT; plaque size; NIHSS; hs-CRP; adverse reactions |
| You L, et al. 2019 [15] | Carotid atherosclerosis with acute cerebral infarction or TIA | 96 | 59.48±3.59 | 6 | Aspirin and atorvastatin | Carotid IMT; plaque Crouse score; hs-CRP; MMP-9 |
| Sun L, et al. 2021 [13] | Carotid atherosclerosis with acute cerebral infarction | 100 | 58.93±2.17 | 3 | Antiplatelet aggregation and lipid-lowering agents; thrombolysis | Carotid IMT; plaque size; plaque Crouse score; NIHSS; adverse reactions |
| Li S, et al. 2011 [16] | Carotid atherosclerosis with hypertension | 60 | 70-86 | 6 | Aspirin and antihypertensive agents | Carotid IMT; hs-CRP; adverse reactions |
| Wang S, et al. 2017 [17] | Carotid vulnerable plaques with acute cerebral infarction or TIA | 100 | 40-79 | 6 | Aspirin and atorvastatin | Carotid IMT; plaque Crouse score; hs-CRP; TNF-α; MMP-9; adverse reactions |
| Wang Y, et al. 2021 [18] | Carotid atherosclerosis with acute cerebral infarction | 200 | 54.69±5.16 | 6 | Aspirin and atorvastatin | NIHSS; hs-CRP; TNF-α; adverse reactions |
| Qiu Y, et al. 2018 [19] | Carotid atherosclerosis with acute cerebral infarction | 102 | 53.5±11.4 | 1 | Antiplatelet aggregation and lipid-lowering agents; edaravone | Carotid IMT, plaque size and NIHSS |
| Lin Y, et al. 2022 [20] | Carotid atherosclerosis with acute cerebral infarction | 60 | 67.1±3.8 | 12 | Antiplatelet aggregation and lipid-lowering agents | Carotid IMT; NIHSS; adverse reactions |

IMT: intima-media thickness; NIHSS: National Institute of Health Stroke Scale.

**Supplementary Fig. 1.** Quality assessment of the included RCTs. (A) Risk of bias graph: each risk of bias item presented as percentages across all included studies. (B) Risk of bias summary: each risk of bias item for each included study.


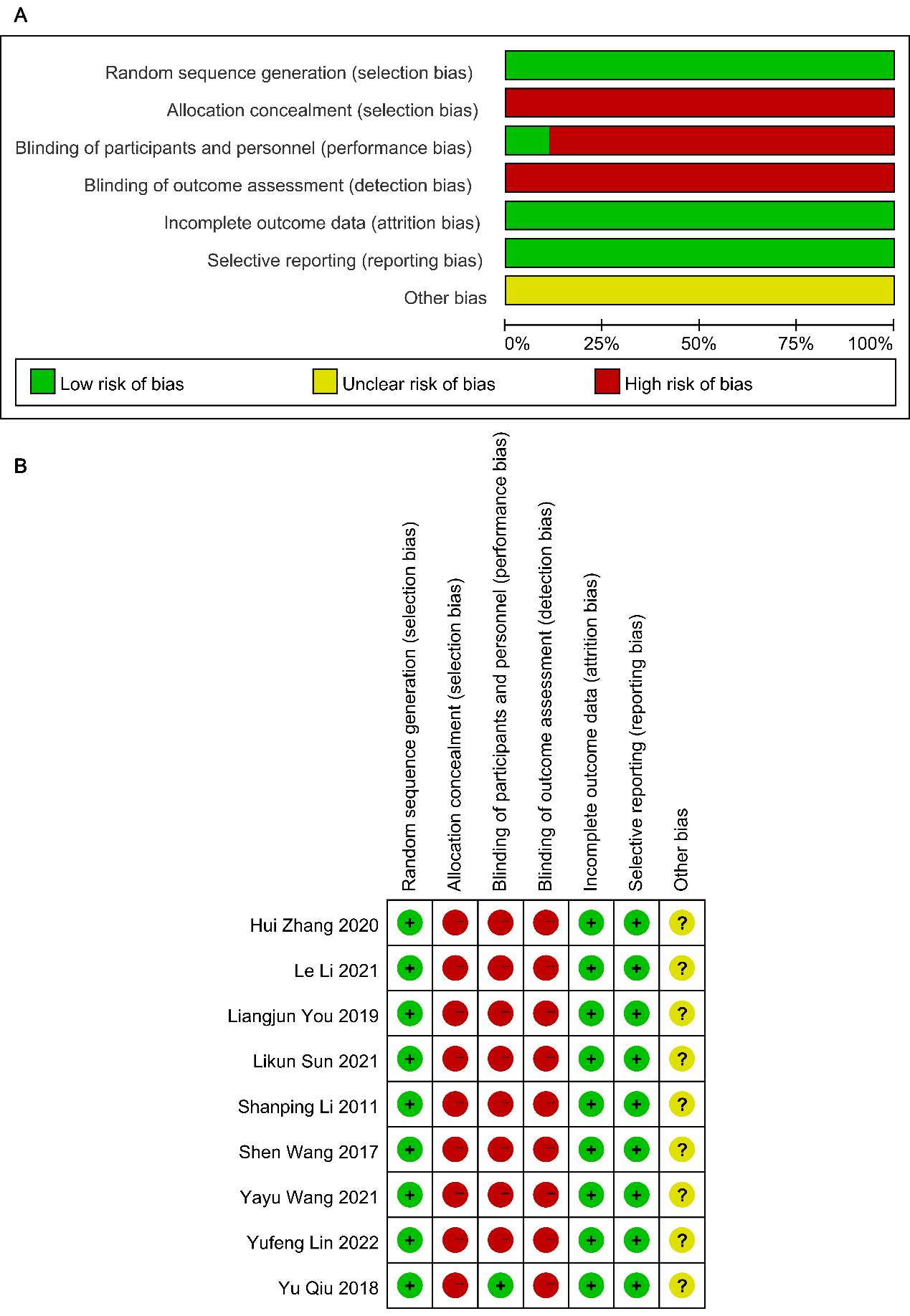


**Supplementary Fig. 2.** The pooled effects of butylphthalide on circulating hs-CRP and MMP-9 levels. (A) Effect of butylphthalide on circulating hs-CRP level; (B) effect of butylphthalide on circulating MMP-9 level.


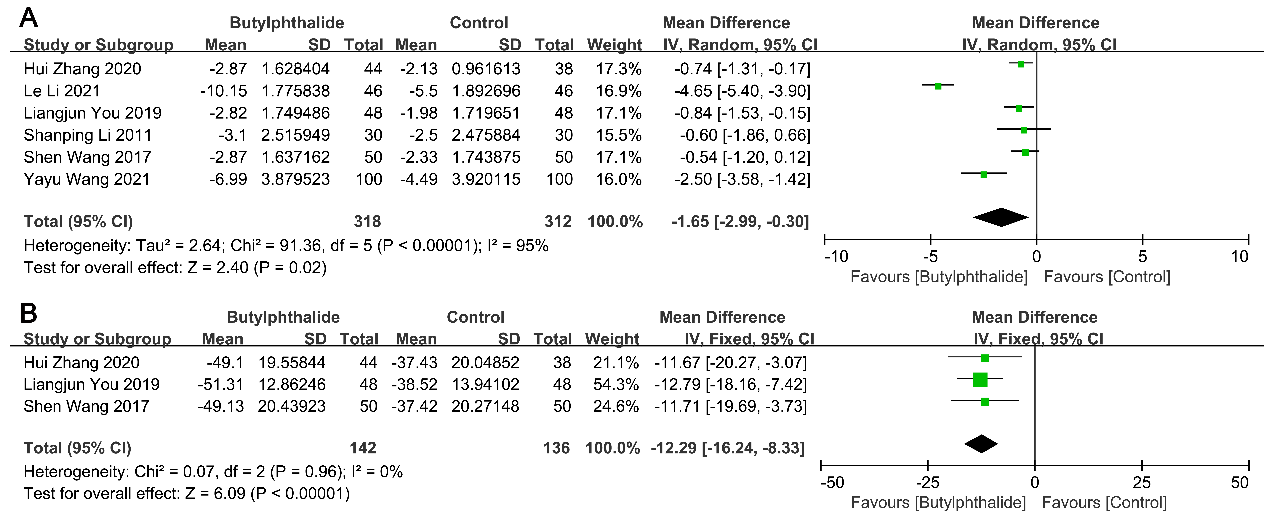


**Supplementary Fig. 3.** The pooled effect of butylphthalide on drug-related adverse reaction.


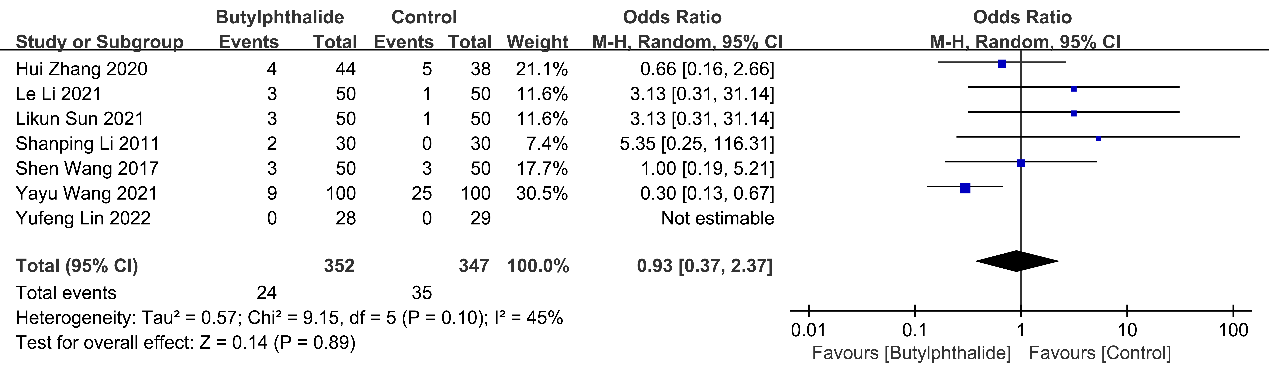

Supplement: Supplementary file 1 [file DataSheet1.docx]
